# Pedagogical applications of cognitive research on musical improvisation

**DOI:** 10.3389/fpsyg.2015.00614

**Published:** 2015-05-11

**Authors:** Michele Biasutti

**Affiliations:** Department of Philosophy, Sociology, Education and Applied Psychology, University of Padova, Padova, Italy

**Keywords:** musical improvisation, cognitive models, cognitive processes, pedagogy on processes, music creativity

## Abstract

This paper presents a model for the implementation of educational activities involving musical improvisation that is based on a review of the literature on the psychology of music. Psychology of music is a complex field of research in which quantitative and qualitative methods have been employed involving participants ranging from novices to expert performers. The cognitive research has been analyzed to propose a pedagogical approach to the development of processes rather than products that focus on an expert’s use of improvisation. The intention is to delineate a reflective approach that goes beyond the mere instruction of some current practices of teaching improvisation in jazz pedagogy. The review highlights that improvisation is a complex, multidimensional act that involves creative and performance behaviors in real-time in addition to processes such as sensory and perceptual encoding, motor control, performance monitoring, and memory storage and recall. Educational applications for the following processes are outlined: anticipation, use of repertoire, emotive communication, feedback, and flow. These characteristics are discussed in relation to the design of a pedagogical approach to musical improvisation based on reflection and metacognition development.

## Introduction

The study of musical improvisation has attracted the attention of many researchers in recent years. Several empirical studies have been carried out, and various theoretical models have been proposed (Pressing, 1988; Johnson-Laird, 2002; Kenny and Gellrich, 2002). These studies have developed appreciably since the 1980s based on increased interest in the understanding of jazz improvisation. Improvisation research has developed in many directions and has employed both quantitative and qualitative approaches involving participants ranging from novice to expert performers. Improvisation has also been analyzed from different perspectives in relation to fields such as ethnomusicology, musicology, psychology, and education. In ethnomusicology, the characteristics and practice of musical improvisation in various cultures are considered (Nettl, 2009). In musicology, stylistic variables and the contribution of the individual author as well as the historical evolution of improvisation styles are analyzed (Givan, 2014). In psychology, the cognitive processes involved in musical improvisation and the interactions and communication dynamics between performers are studied (Pressing, 1988; Johnson-Laird, 2002; Kenny and Gellrich, 2002; Sawyer, 2011). In education, the conditions for skills development are analyzed by highlighting the best methods for teaching and learning improvisation (Beegle, 2010).

Although several fields of research can be considered in developing educational models, the current review primarily takes into account cognitive psychology studies. The aim of this research is to implement an educational approach to the development of processes rather than products that focus on an expert’s use of improvisation. Some leading questions in this review include, “What are the characteristics of improvisation?”; “What are the differences between improvisation and composition?”; “What are the main psychological processes underpinning musical improvisation?”; “What are the skills and competences involved in musical improvisation?”; “How has musical improvisation been taught?”; “What principles can be used to develop an improvisation teaching approach based on the development of processes?”; and “What role and function does improvisation have in music education?”

## Definitions of Improvisation

Several definitions have been proposed that focus on specific aspects of improvisation (Azzara, 2002; Hickey, 2002; Cambridge, 2015; Merriam-Webster, 2015). Some current definitions drawn from dictionaries and encyclopedias underscore the compositional dimension and the immediacy of the creative expression of improvisation, including composing extemporaneous verses, inventing music without previous preparation, creating music in real-time or “making or creating (something) by using whatever is available” (e.g., Merriam-Webster, 2015). Other definitions, such as composing and performing simultaneously, note the multiplicity involved in improvisation, although several studies have shown that the composition process has different dynamics compared with improvisation (Sarath, 1996; Azzara, 2002). Some other definitions stress the performance dimension and unique levels of improvisation, such as impromptu performances that are completely left to the imagination of the instrumentalist, whereby the performer is free to express his personality, or “a performance that an actor, musician, etc. has not practiced or planned” (e.g., Cambridge, 2015).

Other definitions include both the ability to perform impromptu music in a creative and spontaneous way and the issue of respecting certain principles. Azzara (2002, p. 172) notes, “improvisation means that an individual has internalized a music vocabulary and is able to understand and to express musical ideas spontaneously, in the moment of performance.” Hickey (2002) considers the relevance of cognitive components that are developed in a social context. These definitions consider the complexity of the processes and abilities involved during improvisation. However, some authors claim that music creativity is a skill that is potentially present in all people, and at a basic level, untrained musicians and children have been involved as participants in the research (Burnard, 2012). Hewitt (2008) believes that musical creativity is a trait that could be expressed by any person in a collaborative composition. Conversely, in expert improvisations, technical abilities and the grammar of music are involved. The skills required to improvise are frequently investigated as a continuum, but it is difficult to distinguish where the expert level starts on this continuum.

Despite the different definitions and approaches, there are some recurrent aspects of improvisation. It can be argued that improvisation is a key aspect of musical expression that has been used in different forms and based on different principles. Improvisation can be found in most cultures and ethnic groups, and it could be used as a tool to assess the complexity of the culture that produced it (Nettl, 2009). In addition, there are historical and stylistic variables involved because the technique of improvisation occurred at different times in music history. Improvisation does not occur randomly, but in relation to a framework that guides and defines the choices of performers (Azzara, 2002). Musical improvisation involves musical and social norms that must be respected (Berliner, 1994). To better understand the characteristics of improvisation, it is useful to delineate the differences between improvisation and composition.

## Differences between Improvisation and Composition

Several studies have considered the similarities and differences between improvisation and composition (Sarath, 1996; Azzara, 2002; Lehmann and Kopiez, 2010). The issue is defining whether these practices are characterized by qualitative or quantitative differences in the nature of the processes involved. The various definitions and models articulate that improvisation is the real-time creation and performance of music, while composition involves reflective processes without the pressure of instantaneously producing the music piece. Compositional processes can be developed using dynamics that are not necessarily linear, which creates a complex framework. Such is the case with the application of theories taken from other disciplines such as math, physics and science, as occurred in the twentieth century. During composition, the musical material can be developed and reworked at any time with maximum control. The score is the output of the composition process, and it can be assessed and changed to correct mistakes or introduce improvements. Conversely, improvisation is an irreversible action; it is not possible to stop a performance, go back and review. However, during improvisation, it is possible to modify the forthcoming performance: improvisation has an adaptable and interactive nature, and a performance could be modeled and adjusted in real-time.

According to Sarath (1996), improvisation and composition are developed in different contexts and involve specific cognitive skills: improvisation is a single and continuous act, while composition is a discontinuous act based on the possibility of revising the score. Composition allows one to design music pieces with a high level of complexity, which is difficult to achieve with improvisation, or at least to have the complete control over all of the operations performed. These dynamics are particularly evident when composing music pieces for large ensembles or orchestras in which the development of all of the instrumental parts can be carefully monitored. Conversely, during improvisation, musical developments can be defined quite precisely when there is only one performer improvising, while when there are more musicians who are improvising simultaneously, the music development depends on the variability introduced by each performer. In ensemble improvisation, the musical framework is outlined by the combination of the unpredictable aspects of the performances, and the context and communicative dynamics determine the variability in the improvisation.

Improvisation is mainly a social activity that takes place among a group of individuals who collaborate to spontaneously produce a coherent piece of music (Monson, 1996; MacDonald et al., 2011). The social aspect is a core feature of improvisation, as noted by Azzara (2002, p. 172): “improvised music relies on the interaction among musicians performing in the moment as compared to a sole composer making musical decisions outside of real-time.” Musicians use sophisticated communication skills to successfully interact, and sharing and collaborating are important characteristics to effectively develop a musical improvisation (Sawyer, 2006).

Improvisation involves both generative processes and instrumental techniques to produce a complete music piece that contains the creation and interpretation of musical ideas. Conversely, composition includes individual productive processes synthesized in a score, but players are needed to perform the score. The performance of a music piece introduces several additional variables because there could be different levels of engagement and personal idiosyncrasies in interpreting a score. Despite the different processes involved in composition and improvisation, the final output seems to have similar features for the listener. Lehmann and Kopiez (2010) demonstrate that it is very difficult to discern through listening whether recorded music is freely improvised or performed according to a score. In addition, there are relationships between improvisation and composition: improvisation can act as a stimulus to the composition process when composers use improvisation to experiment with new content, and they then select the most interesting material for the music piece (Biasutti, 2012). Improvisation can offer fresh ideas to work on and can provide a touch of originality to a composition. The characteristics of improvisation and composition are reported in Table 1.

**TABLE 1:** **Characteristics of improvisation and composition**.

**Characteristic**	**Improvisation**	**Composition**
Context	Public or private	Private
Individual/group	Individual or group	Individual or group (less frequent)
Development	Continuous, linear, real-time act, extemporaneous creation	Continuous or discontinuous act, mediated creation
Experimentation	Extemporaneous experimentation; it can provide suggestions to composition	Reasoned experimentation
Abilities	Performing and compositional	Compositional
Processes	Anticipation, use of repertoire, emotive communication, feedback, and flow	Planning, translating from the sound to graph, idea generation, organization and construction, revision
Reversibility	Irreversible action, it cannot be changed	Reversible action, it can be changed until the final draft
Revision	It cannot be reviewed, but only adjusted in real-time during the performance with the feedback	It can be reviewed and improved
Control	Control of individual variables but not of group variables	Overall control of the score and of the complexity of the compositional process
Feedback	Real-time feedback	Feedback without real-time pressure
Process dynamics	Interactive process. It has an adaptable nature, it allows you to answer to context variables, it can be adjusted instantly. Challenge between performers, taking risks	Fix product. The composition can be interpreted but it is not possible to change the notes of the score
Communication	The author has a direct communication with the audience. It is more authentic and real than composition	The author has a communication with the audience mediated by the performer(s) who interprets his/her ideas

In conclusion, substantial differences in the processes of composition and improvisation have been highlighted: improvisation has a more versatile nature during a performance, and it can be adapted more easily to different circumstances than composition. The adjustment involves feedback processes in which responses to context variables are produced: musical events are tuned according to the various situations that arise. Conversely, composition is flexible during the composing process because the composer is free to define all of the details of the score. However, when it is defined, the score is a fixed framework that must be respected by the performer. In discussing the differences between improvisation and composition, several cognitive aspects have emerged that have been analyzed in psychological studies on musical improvisation.

## Psychological Models of Musical Improvisation

There is growing interest in the psychological processes of musical improvisation, which is considered a complex area of research. Beaty (2015, p. 109) argues that improvisation is one of the most articulated expressions of creative behavior: “The improvising musician faces the unique challenge of managing several simultaneous processes in real-time—generating and evaluating melodic and rhythmic sequences, coordinating performance with other musicians in an ensemble, and executing elaborate fine-motor movements—all with the overall goal of creating esthetically appealing music.” This description notes that improvisation consists of a conglomeration of mental and motor processes that result in the real-time generation of music. Several constructs have been studied in prior research, such as how working memory capacity relates to musical improvisation (De Dreu et al., 2012), the activation of particular states of consciousness (Csikszentmihalyi, 1990; Csikszentmihalyi and Rich, 1997), conceptions of learning and performing musical improvisation (Biasutti and Frezza, 2006), shared understanding (Schober and Spiro, 2014), and emotional and expressive aspects (Gilboa et al., 2006; McPherson et al., 2014). Several models have been developed to attempt to explain the underlying decision-making mechanisms in idea generation and feedback during musical improvisation (Pressing, 1988; Johnson-Laird, 2002). Several issues are faced in developing such models, including how new material is generated, how the processes are coordinated and how expertise is developed.

Regarding how new material is generated, Norgaard (2011) conducted an interview study about the improvisational thinking finding the following four strategies used by seven jazz musicians for generating the note content of the improvisations: recalling well-learned ideas from memory and inserting them into the ongoing improvisation, choosing notes based on a melodic priority, choosing notes based on a harmonic priority, and repeating material played in earlier sections of the improvisation. Regarding how the processes are coordinated, Pressing (1988) describes the functioning of cognitive structures for production and control during improvisation and highlights the centrality of generation and feedback in decision making. Regarding generation, music events are produced by mental processes that include an idea about the sound and the related motor behavior. Feedback is a monitoring process used by performers to contrast their purposed output with the real event produced. Short-term feedback, which concerns the actual motor behaviors, can be differentiated from long-term feedback, which involves decision making and the selection of actions. These processes are useful for adjusting a performance and are crucial for achieving fluency during improvisation.

Johnson-Laird (2002) notes that the primary driving forces of improvisation are certain rules that are used to select the notes and the musical material. The issue is identifying a grammar and a set of rules for improvising. According to this approach, information processing is performed in a similar manner for all subjects, and an expert improviser is distinguished by the quality and richness of the grammar used. An expert improviser has internalized the grammar whereby the decision-making process is implicit, with little involvement of the working memory.

Pressing (1988) argues that improvisational expertise relates to the interaction between a set of mechanisms and the knowledge base. The mechanisms within which improvisation is created are perceptive, cognitive, and emotional processes, while the knowledge base represents hierarchical knowledge structures that are stored in the long-term memory. The mechanisms relate to procedural and declarative knowledge stored in the knowledge base to produce a musical improvisation. Through the practice of improvisation, a database of schemes and motor programs is developed that can be used during a performance. Preformed structures stored in procedural memory are inserted during improvisation (Norgaard et al., 2013). Improvisation is acted on in real-time, and all of the actions require information processing, which can be coordinated and accelerated to obtain a fluent performance. During practice, low-level motor and cognitive processes are automatized, which allows the cognitive resources to be focused on higher-order cognitive processes.

Pressing’s (1988) model is used as a reference to interpret the results of neuroscience research on improvisation (Beaty, 2015). Beaty (2015) summarizes the results of neuroimaging studies by noting that a network of prefrontal brain regions is usually associated with improvising behavior, which assumes cooperation between large-scale brain networks, cognitive control and spontaneous thought. Beaty (2015) argues that Pressing’s (1988) model fits quite well with the data from neuroimaging studies. However, the extent to which activation patterns reflect theoretical mechanisms remains unclear.

The reviewed models have highlighted the complexity of improvisation, which is considered a multidimensional act that involves sensory and perceptual encoding, motor control, performance monitoring, and memory storage and recall. Several processes have been considered in the models, such as idea generation and feedback and the importance of developing a set of rules and an improvisational grammar. However, the overall competences involved in musical improvisation are not completely defined.

## Competences and Expertise in Improvisation

Regarding the competences and the expertise involved in improvisation, two primary studies define an overall framework of the knowledge and skills required for improvisation (Biasutti and Frezza, 2009; Wopereis et al., 2013).

**FIGURE 1:**
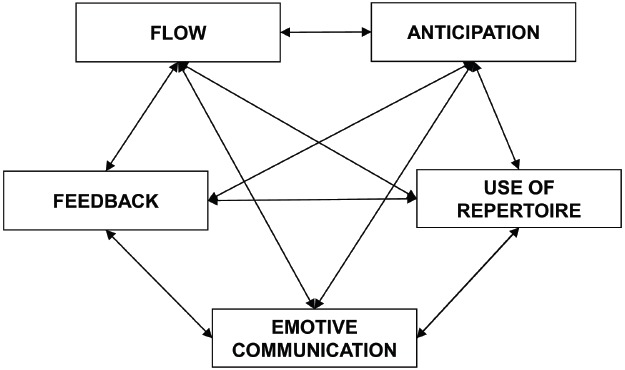
**The dimensions of musical improvisation by Biasutti and Frezza (2009)**.

Biasutti and Frezza (2009) consider the processes involved during musical improvisation and propose a model that includes the following five dimensions: anticipation, use of repertoire, emotive communication, feedback, and flow (see Figure 1). A quantitative approach is employed to test the model using adult musicians who are administered the Improvisation Processes Questionnaire. Exploratory factor analysis confirms the extraction of the five factors noted above. The anticipation factor refers to the ability to anticipate features and characteristics at the rhythmic, melodic, and harmonic levels that correspond to musical events that must be performed. The use of repertoire factor concerns patterns such as licks, or clichés, that musicians commonly use during improvisation. The emotive communication factor is related to the skill of communicating emotions through improvisation. The feedback factor involves the monitoring processes by which changes and adjustments to improve a performance are made in accordance with the relevant information collected about the performance. The flow factor refers to a state of mind that includes cognitive, affective, and physiological aspects that are linked to flow or an optimal experience.

Wopereis et al. (2013) use a group concept mapping procedure to determine the characteristics of improvisational expertise among skillful musicians. Multivariate analyses are used to process the data, resulting in a 7-cluster concept map that includes the following clusters: self-regulation, basic (musical) skills, affect, risk-taking, creation, responsivity, and ideal. The self-regulatory cluster is central to the map. This cluster focuses on the knowledge and skills needed to start, go through, and end an improvisation and includes statements related to connecting musical ideas, playing and not playing, and anticipation. The basic skills cluster refers to skills and attitudes that are elementary to improvisation. The affect cluster includes statements that allude to the emotions and feelings experienced during a musical improvisation. The risk-taking cluster concerns statements that refer to the management of personal and musical constraints. The creation cluster is characterized by statements related to instantly organizing, generating, and composing music. The responsivity cluster comprises statements that emphasize the interactive nature of improvisation. The ideal cluster highlights statements related to perceived idealized improvisational expertise.

These two studies provide an idea of the overall competences involved in improvisation, and they highlight the centrality of anticipation (Biasutti and Frezza, 2009) and self-regulatory behaviors (Wopereis et al., 2013). Several processes are considered in both studies, including anticipation, emotive communication, and the use of the repertoire and feedback, although the concepts are labeled with different terms.

## Models for Teaching Improvisation

Kratus (1991, 1996) proposes a model of improvisation pedagogy based on the cognitive research linking spontaneous and intuitive with expert and musically refined behaviors. This developmental teaching approach considers music improvisation “as being multileveled, consisting of a sequence of different, increasingly sophisticated behaviors” (Kratus, 1991, p. 38). A seven-level sequential model has been theorized which is independent of age, and the musical features are indicative of a specific level of improvisational skill. The educational activities depend on the students’ level of knowledge and skills. The first level, exploration, could be considered as a pre-improvisational activity and consists of making random sounds without audiate them. At this and at the following level the teacher’s role is to teach to audiate, providing students sufficient time and a variety of sound sources for exploration. At the second level, process-oriented improvisation, “once a student begins to audiate the patterns played in exploration, the resulting music becomes more directed and pattern dominated” and some micro-structures emerge (Kratus, 1991, p. 38). The third level, the product-oriented improvisation, is acquired when “the student’s improvisations begin to show such characteristics as the use of a consistent tonality or meter, the use of a steady beat, the use of phrases, or references to other musical pieces or stylistic traits” (Kratus, 1996, p. 33). At this level the teacher’s role is to provide students with different constraints on their improvisation, such as to give as certain rhythm patterns or set of chord changes to improvise on. The fourth level, fluid improvisation, starts when the performer acquires adequate technical control of the instrument so that the technical manipulation becomes automatic, and the musical ideas are more easily transformed into sound. At this level the teacher’s role is “to focus on the technical facility by providing the student opportunities to improvise in a variety of modes, keys, meters, and tempos” (Kratus, 1991, pp. 38–39). The fourth level, structural improvisation, emerges when the performer develops an awareness of the overall structure of the improvisation and can apply structural techniques for shaping an improvisation. At this level the teacher’s role is “to introduce the student to different musical and non-musical means for connecting musical ideas and structuring an improvisation” (Kratus, 1996, p. 35). Music analysis of other solos is used for deducing models of organization and strategies for developing musical ideas are provided. The sixth level, stylistic improvisation, emerges when the improviser has mastered one or more improvisational styles and has learned the characteristics which define the style. At this level the teacher’s role is to help students to “develop a performance repertoire of the specific rhythms, melodic patterns, harmonic characteristics and timbral qualities that serve to identify a given style” (Kratus, 1996, p. 35). The seventh level, personal improvisation, emerges when the improviser transcends current styles to develop a new and original style. At this level the teacher’s role is to encourage the student to acquire competency in a broader range of styles, because sometimes new styles of improvisation emerge when features of diverse styles are merged.

The strengths of this model are to define the improvisation along a continuum without fractures between one level to the other and to start teaching improvisation at a very basic level. Probably there could be an overlapping between some levels, because the organization dimension which characterizes the structural improvisation, can be introduced also before, because within fluid improvisation you can get an idea of the development and structure of the improvisation. The model considers the development of several skills including listening and the concept of audiate, but it does not address all the specific processes involved in music improvisation with a process oriented teaching.

## A Model for Teaching Musical Improvisation through Processes

Regarding applications of the cognitive research on improvisation in education, the literature review provides a complex framework that can be used to reflect on the processes involved during improvisation. These reflections provide several inputs to define a teaching approach based on the development of processes rather than products (Biasutti, 2013b). This approach can be proposed at several levels including adolescents and adults in music conservatories and academies. A process-oriented teaching method can assist the acquisition of strategy-enhancing skills such as problem solving and critical thinking and can promote a reflective practice regarding the processes involved during improvisation.

The intention is to delineate a reflective approach that goes beyond the mere instructional level of some current practices of teaching improvisation in jazz pedagogy. In the past, learning jazz was an everyday process that was developed through informal learning contexts and performances (Berliner, 1994). Since its introduction into formal courses of study, academic jazz programs have defined precise curricula using instructive models. A music-theoretical approach based on scales, arpeggios, chords, patterns, and harmonic progressions that informs jazz improvisation was proposed (Huovinen et al., 2011). Several manuals tried to standardize the teaching and learning processes of jazz by proposing instructive models for acquiring the grammar of musical improvisation, but they neglected the jazz tradition and the role of informal learning. Playing by ear is underexposed in the current approaches, which stress notated instruction and exercises such as scales, chords, and scale/chord associations. Huovinen et al. (2011) argue that most of the approaches in the improvisation pedagogy focus on basic skills development. Improvisation is often taught using separate rhythmic, melodic and harmonic exercises, and jazz standards are used as the most relevant educational materials. The instructor teaches jazz theory and uses models that provide musical examples of improvisation. Hickey (2009) notes that one of the most common approaches is the “riff” approach, which consists of memorizing jazz patterns and reproducing them over several chord changes. Other approaches include tasks such as listening and transcribing solos, practicing with minus one recordings, sight reading, instrumental and vocal technique training, and arranging and composing activities. In addition to theoretical knowledge and scale and chord practice, more innovative proposals include activities such as aural skill development, playing by ear and providing an authentic learning environment in which opportunities to play with more experienced improvisers are offered. Azzara (1993) proposes a sequence of educational activities to promote improvisation learning that includes (1) learning a selected repertoire of songs by ear; (2) developing a vocabulary of tonal syllables in major and minor tonalities and rhythm syllables in duple and triple meters; (3) improvising vocally and instrumentally tonic, dominant, and subdominant tonal patterns within the context of major tonality; (4) vocally and instrumentally improvising macrobeat, microbeat, division, elongation, and rest rhythm patterns within the context of duple meter; and (5) improvising a selected repertoire.

A key issue in learning instrumental improvisational is proposing an approach to developing awareness of the skills involved in improvisation rather than an approach that is centered on instruction (as occurs in improvisation training using separated scales, arpeggios, and chords exercises). It would be interesting to revitalize the tradition of free expression in improvisation (Borgo, 2007; Hickey, 2009) by focusing on a combination of technical and expressive aspects to develop a complete understanding of music as a language.

In a pedagogical approach on processes, the teacher focuses on the development of the actions that are necessary for improvisation. Thus, the processes that are involved in improvisation are identified to determine the relevant educational activities. The strengths of a teaching approach that is focused on the processes are that individual processes can be fortified and connected in a holistic framework to support the overall implementation of an improvisation. Process-oriented teaching requires a greater workload in the designing phase—in which specific activities are defined—than traditional approaches. In addition, the teacher assumes the role of facilitator to enhance the students’ awareness during improvisation rather than imposing notions and theories on the students. Thus, it would be interesting to discuss the educational implications of a process-oriented model based on the cognitive research in relation to the five dimensions highlighted by Biasutti and Frezza (2009): anticipation, use of repertoire, emotive communication, feedback, and flow. These aspects have been scrutinized individually in educational studies, but an organic approach would be beneficial to coherently develop the processes involved in improvisation. The process-oriented approach is transferable and could be applied to the pedagogy of several different instruments and music genres.

### Anticipation

Anticipation means thinking ahead about features and characteristics at the rhythmic, melodic, and harmonic levels during musical improvisation. It refers to the ability to foresee, in a relatively accurate way, the objects, patterns, phrases, and process arrays that correspond to the musical event clusters that are to be played (Pressing, 1988; Kenny and Gellrich, 2002). Anticipation is a fundamental aspect of idea generation, and it affects the overall quality of musical improvisation by influencing the decision-making process. It is correlated with the real-time dynamics of improvisation and the ability to instantaneously generate musical ideas. Anticipation is a logical activity that involves a cognitive effort, and it facilitates the improviser in finding complex solutions at the melodic, rhythmic, and harmonic levels. Anticipation relates to the ability to plan sound events in the sequence that is required for the performance. Thus, the generation of fresh ideas is combined with the ability to define a framework (De Dreu et al., 2012). Musical sequences are considered as a gestalt rather than single tones, and the musical material is articulated in clusters (Pressing, 1988). Anticipation involves the skill of gaining an extensive idea about an entire solo and designing an improvisation. It concerns the definition of a general plan that allows one to collocate needed musical material in the right place during a musical improvisation.

There are several educational activities that could be focused on anticipation, such as planning an improvisation and defining the aspects of the context of a performance. A plan provides a reference for the generation of ideas, and having a plan in mind facilitates the anticipation process. The musical events of an entire solo could be outlined, and a music practice could include stopping a performance to think aloud and describe the development of an improvisation. Other activities include singing the melody one is playing to oneself (Biasutti and Frezza, 2009). This technique is quite common in musical improvisation practice where students are stimulated to anticipate musical events and the melody to be played.

### Use of Repertoire

The use of repertoire concerns the employment of formulas and patterns such as licks^1^ during musical improvisation. Licks is a term used for denoting short musical motifs. Musicians often use prelearned melodic and rhythmic figures as a basis for developing an improvisation, and this repertoire is stored in long-term memory. Licks are defined according to the music genre performed and are often deduced through the study of the solos of famous musicians. To broaden a learner’s vocabulary, many pedagogical approaches include learning tasks that consist of extensive reviews of recorded solos that are exemplary to various musical styles. Listening to these recordings stimulates learning by ear. There are several steps that can be followed. It starts with the memorization of solos, followed by learning to sing them and finally playing them on a musical instrument. The first step involves the auditory system and the perception and identification of the licks. The second step involves singing and playing them on an instrument, which also develops a motor component because the lick is learned as both an auditory mental representation and the corresponding motor schema. The cognitive research literature highlights the importance of both aural and motor schema (Pressing, 1988; Beaty, 2015). However, during idea generation, it is not clear whether music licks are selected considering aural cues or motor cues or both.

The “riff” approach considers the memorization of patterns and their extensive use during improvisation (Hickey, 2009). This pedagogical approach relates to the belief that improvisers use licks to generate solos. This approach—which is based on analyses of improvisations of famous performers—allows the learner to widen the long-term memory database about the licks to be used for improvisation. However, it is relevant to articulate the educational framework, which considers several levels, such as declarative knowledge, procedural knowledge, and situated knowledge. The basic level is knowing the licks (declarative knowledge), but it is also relevant to know the procedures and the processes about how to use them (procedural knowledge) in relation to a specific situation (situated knowledge). Using procedural knowledge diminishes the cognitive load of the task and contributes to making the improvisation process more efficient. Johnson-Laird (2002) notes that it is crucial to obtain a grammar to improvise, and it is relevant to teach strategies about how to use musical material. Educational activities need to be focused on the acquisition and application of principles that can be used to work on licks. Students should be provided with strategies for selecting and elaborating on licks.

It is relevant to offer a learning environment that is rich with stimuli that exemplify the possible utilization of the basic material and provides strategies for using licks. An additional activity involves a task analysis to determine all of the actions included in the elaboration of licks. This process leads to step-by-step instructions needed to complete a job. The aim is to specify educational activities that go beyond the instructional level of music pattern repetition to promote independent thinking. Students are asked to produce variations and to introduce original aspects in re-elaborating famous solos. It is relevant to delineate a creative environment in which predefined musical patterns are used to develop idea generation and performance techniques. The licks must be reprocessed according to a different music style, thus accounting for the creative dimension of improvisation. A divergent utilization of licks should be involved in which the musician experiments with new solutions. The aim is to promote a reflective practice about the use of licks by thinking about strategies to be used in relation to a certain musical context. Improvisers not only need knowledge of the repertoire but also of the rules for using it, and they must develop a consciousness of the strategies adopted.

### Emotive Communication

Emotive communication refers to the transmission and induction of emotional and affective states during musical improvisation. It is a process that allows one to express certain inner feelings. Musical communication and expression are conducted through musical language, stylistic principles, and the modulation of relevant sound parameters such as rhythmic, melodic, harmonic, dynamic, and timbre dimensions. Emotive communication is connected with knowledge of musical grammar because the greater the number of known formulas is, the more sophisticated are the concepts that can be expressed. During improvisation, idea generation is also mediated by emotions and other inner feelings. McPherson et al. (2014) examine the structural features of free musical improvisations generated by jazz pianists as a consequence of emotional cues. The findings demonstrate a lack of simple correspondence between the emotions and musical features of an improvisation. The performers combine heterogeneous musical characteristics to describe positive and negative emotions rather than using universal patterns to convey emotions. The study provides evidence that structural diversity may be an essential feature of the ability of music to express a wide range of emotions.

Educational activities on emotive communication include tasks such as asking students to improvise based on a specific feeling. These activities offer students the opportunity to combine disparate features to express inner states during improvisation. In addition, activities such as verbalization of the emotions conveyed during improvisation associated to the musical events performed could induce a reflective practice. Emotive communication should be brought to the conscious level in which awareness of the communicative dimension and the process of expressing inner states is raised.

Working on emotive communication could be a way to provide students with more meaningful activities because when expression is regarded as more important than notes, students are more involved and interested. Parncutt (2006) notes that when the semantics—the meaning of music—are conceived as more important than the syntax (the melodic, rhythmic, and harmonic musical structures), the syntactic patterns paradoxically become easier to learn.

### Feedback

Feedback refers to the ability to react to certain phenomena by making real-time changes to synchronize or improve an improvisation. It allows one to modify an improvisation instantaneously to make it more consistent and coherent in various situations. Feedback is a point of reference for the development of the ability to improvise, and it specifically involves monitoring processes: performers contrast their intended performance with the real event produced. Feedback can be extremely varied in relation to the characteristics of the context, the music, and group dynamics. Performers adopt a variety of strategies and modalities for using feedback during performances, and several types of feedback have been identified, such as short-term and long-term feedback (Pressing, 1988) and internal and external feedback (Biasutti and Frezza, 2009). Pressing (1988) defines short-term feedback as a process that is needed to monitor actual motor behaviors, while he describes long-term feedback as a process that is involved in decision making and the selection of actions. Biasutti and Frezza (2009) consider internal feedback to be the process used by musicians to monitor their performances and external feedback as the information exchange between performing musicians, the audience and the environment. Feedback depends on the characteristics of the musical environment (e.g., rhythm, melody, and harmony) and behavioral modification and regulation. Musicians consider different formats of information in internal feedback, such as auditory and proprioceptive cues. External feedback between performers could be based on events such as gestures (where musicians use gestural communication and conventional signals to determine the order of improvisation, attacks etc.), visual cues (where musicians look at each other’s facial expressions), and musical cues (where musicians respond to each other in an atmosphere of musical dialog and use musical cues to inform their mates about the development of the piece).

Educational activities on feedback should improve musicians’ ability to react instantaneously in different ways to the various inputs that can occur during an improvisation. Feedback is helpful for controlling and managing problematic situations that may emerge and can be a stimulus to differentiate musical communication and develop a music discourse. Several feedback exercises could be proposed, such as musical question and answers, which could be performed with eyes closed to enhance the focus on the qualities of the sound. This activity could induce musical dialogs based on several parameters, such as rhythm, melody, harmony, and timbre. Other tasks could provide musical ideas that change abruptly and unexpectedly, and students are asked to follow these changes while improvising. Giving cues through verbal communication about how to improvise could be another technique for training the ability to react in real-time during improvisation. The verbal commands could be changed frequently to induce a different tone of the phrasing in a performance. Providing diversified cues for developing improvisation such as visual stimuli (e.g., a picture or an image), aural stimuli (e.g., a word, a sound, or a noise), or kinesthetic stimuli (e.g., a movement or a gesture) expands the synesthetic creativity of the improvisers: music is interpreted through inputs from different sensorial modalities. The principles that can be used while improvising are to follow the character of a proposal or to fill it in opposition.

An advanced use of group feedback can activate risk-taking mechanisms when musicians challenge each other (Wopereis et al., 2013). The participants engage each other in challenging musical interactions to produce original variations during idea generation. The final output is unpredictable because of multiple levels of indeterminacy and the possible combinations of the interactions. Risk-taking is particularly significant in group performances because of the interactions between members. Students should be aware of the importance of feedback and should develop the skills to master it and use it in a proficient manner.

### Flow

Flow is a state of mind in which people are completely focused on an intriguing activity. It involves complete absorption in an improvisation and the comprehensive sensation of operating with total involvement. It includes cognitive, affective and physiological aspects, and is considered an important component of personal well-being (Csikszentmihalyi, 1990; Croom, 2012, 2015). Flow corresponds to a positive experience and poses improvisation activities as pleasant. An individual’s ability to enter into a flow state can be a relevant condition to enhance the individual’s well-being, and it affects the quality of his experiences (Csikszentmihalyi and Rich, 1997). Flow can encourage the practice of improvisation and facilitate the processes of improvisation with a sense of fluency and spontaneity. Flow is connected to optimal experience and peak performance. Compared with the previously described concepts, flow is likely the most comprehensive process. It is a complex, multidimensional construct that includes the following nine aspects: challenge and skill balance, the merging of action and awareness, clear goals, unambiguous feedback, concentration on the task, sense of control, loss of self-consciousness, a distorted sense of time, and an autotelic experience (Biasutti, 2011).

Educational studies have acknowledged the importance of flow state as a concept in teaching (Custodero, 2005; Biasutti, 2011). Custodero (2005), for instance, proposes the following indicators to observe flow: self-assignment, self-correction, gesture, anticipation, expansion, extension, adult awareness, and peer awareness. The relevant studies demonstrate that performance outcomes are improved when educational activities are proposed that assist students in experiencing flow. Regarding activities to encourage flow, the objectives could be focused on creating a performance environment that facilitates flow rather than trying to teach flow directly. Teaching flow directly is complex, while it is effortless to offer opportunities for students to experience flow. In addition, students could be provided with the tools to recognize when flow occurs so they can develop awareness about it.

Basic steps for promoting a flow-friendly learning environment include defining clear expectations and goals (a clear, general objective could be associated with as many sub-objectives as possible) and assessing progress by verifying the achievement of goals. Musical and technical challenges should be combined with existing skills and activities at an appropriate level of difficulty. Relevant strategies include developing the necessary skills involved in an activity and making a task demanding when the activity becomes boring. Providing constructive feedback to students and using feedback to monitor the results of an activity are aspects that provide a real idea about the results achieved. Another condition is to prevent interruptions (Biasutti, 2013a) because they disturb concentration and inhibit the attainment of flow. Strategies that help students concentrate on a task, allow them to become absorbed in a performance, and reduce self-consciousness could enhance flow (Csikszentmihalyi and Rich, 1997). In addition, stimulating the motivation of students and providing the conditions for enjoyment during a performance are relevant aspects that make it easier to attain the flow state (Csikszentmihalyi, 1990).

Group flow is relevant for the quality of outcomes. Group members could take stimuli from flow and become inspired by group interactions to generate a musical product that would not have been possible performing alone. Students can achieve group flow by responding to each other in a positive environment of cooperation that offers the students opportunities to freely express their creativity. Collaboration is an important aspect of group flow: all of the members concentrate on obtaining the same outcome and share goals and objectives. Educational activities on flow are focused on the process level and on the motivation to learn: flow is a dynamic force that can be internalized through direct experiences. Compared with traditional education, flow activities represent an inductive process that is based on a student’s resources and basic abilities rather than on a formal program of learning. The teaching of flow could be a way to innovate in music education by defining a learner-centered approach during lessons.

## Discussion

In the current paper, a teaching approach based on process development was proposed considering the following characteristics: anticipation, use of repertoire, emotive communication, feedback, and flow. A description of the processes and the main supporting educational strategies are reported in Table 2.

**TABLE 2:** **Descriptions and supporting educational strategies of the five improvisation processes**.

**Process**	**Description**	**Supporting educational strategy**
Anticipation	Thinking ahead about features and characteristics at the rhythmic, melodic, and harmonic levels corresponding to musical events	- Defining the context and planning the improvisation - Practicing, including thinking aloud and describing the development of the improvisation - Singing to oneself the melody one is playing
Use of repertoire	Using patterns such as licks or clichés	- Learning by ear - Memorizing, singing, and playing the solos of various musical styles - Analysis of solos - Providing examples of strategies for using licks - Acquisition and application of principles for using licks - Reflective practice on the strategies used
Emotive communication	Communicating emotions through improvisation	- Improvising based on specific feelings - Verbalizing the emotions conveyed during improvisation - Reflective practice on emotive communication
Feedback	Making real-time changes to synchronize or improve an improvisation using monitoring processes	- Musical question and answers - Dialogs based on parameters such as rhythm, melody, harmony, and timbre - Providing improvisational cues through verbal communication - Providing improvisational cues that change abruptly and unexpectedly - Providing improvisational cues based on different sensorial modalities (visual, aural, kinesthetic,…) - Providing challenging and risk-taking musical interactions
Flow	A state of mind of intense absorption during improvisation, including cognitive, affective, and physiological aspects; linked to optimal experience	- Defining clear expectations and goals - Sharing goals and objectives with group members - Defining tasks that are within the participants’ capacity to act - Providing appropriate levels of musical and technical challenges combined with existing skills - Making the task demanding when the activity becomes boring - Assessing progress by verifying the achievement of goals - Providing constructive feedback - Preventing interruptions - Stimulating motivation - Providing conditions for enjoyment

The outlined educational activities define the approach and philosophy of teaching improvisation. Several previously described activities were taken into account in various music education studies with the aim of developing individual abilities. The relevant feature of the current approach is to organically develop the specific and basic processes of musical improvisation by keeping in mind a complete picture of the various processes involved in musical improvisation.

Regarding when improvisation has to be taught, improvisation can be learned at all levels with different aims and purposes, since young children can also express spontaneous improvisational behaviors. Kratus (1996) views improvisation as a variety of different behaviors that develop sequentially and when the students acquire certain types of skills, knowledge and attitudes, they advance to the next level. Several of the proposed activities could be adapted to the levels of the students for developing their basic processes. Another issue regards the relationship between the development of technical skills and the development of improvisation. The improvisational activities could be implemented in a more sophisticated level once a basic level of technical skills has been acquired. A certain level of technical ability allows the performers to better express themselves through improvisation and enable them to develop more refined skills. The development of technical skills and the development of musical improvisation must be considered interrelated rather than separated, since they reinforce each other and are learned concurrently. The issue is to propose an approach which integrates both the technical and the expressive dimensions of improvisation overcoming the barriers of a technical approach as opposed to a musical approach. The aim is to delineate a reflective approach that goes beyond the mere instructional level. A program focused on the processes affects critical analysis and promotes reflection on the task and metacognitive strategies. Students are encouraged to think about their creative processes and to self-assess their experiences, thus developing a more complete awareness about the tasks performed.

This approach is transferable and adaptable in relation to different contents and contexts and could be used in a complementary manner for teaching and learning jazz improvisation. The presented activities should be connected by providing a unified plan for process development. The educational activities should develop technical skills through an intelligible and conscious esthetic expression that considers the overall conception during improvisation. Such activities should be designed to provide a scaffold for students’ learning so they can consider their acts of expression and creativity. In addition, both stylistic characteristics and aspects of musical elaboration such as originality, fluidity, flexibility and processing, which distinguish divergent thinking, must be considered.

## Importance of Improvisation in Education

The importance of improvisational activities in education has been widely discussed by several authors (Azzara, 2002; Sarath, 2002; Hickey, 2009; Parncutt, 2006; Sawyer, 2011) who highlight aspects such as historical relevance, musical skills development, the activation of transfer mechanisms and the role of improvisation in music education.

The importance of improvisation throughout history is noted by Azzara (2002), who shows that improvisation has been an essential component of musical expression. Improvisation disappeared in the nineteenth century with the development of printed music, but until then it was considered common practice to educate students with several sophisticated musical skills simultaneously (Parncutt, 2006). Historical and stylistic aspects are currently used to define a framework for practicing improvisation and developing musical abilities.

Improvisation is considered one of the most integrative musical practices, and it develops students’ rhythmic, melodic, harmonic, and expressive abilities. Improvisation includes perceptual, compositional, and performance skills incorporated through the real-time invention of sound sequences. Improvisation promotes integrated musical development and can be used to educate students at any level. Parncutt (2006) considers improvisation to be an essential practice that is necessarily part of the experience of all musical performers. Improvisation is a formative activity that allows individuals to express themselves and develop higher order abilities in a broad musical context (Azzara, 2002).

Improvisation is considered an activity that induces the transfer of learning mechanisms and can contribute to the development of several related skills in other domains, such as language. Improvisation helps one develop a greater understanding of the relationship between music performed with and without a score. McPherson (1993) notes a transfer process that is activated by improvisation and that stimulates a greater understanding and awareness of the performance involved in reading a score. Music reading could become a passive process in which a performer refers only to a written score and pays little attention to music communication and the qualities of the sound produced. Conversely, improvisation challenges the performer to tackle issues by offering the opportunity to undertake sophisticated problem-solving processes in tasks that imply an ability to react in real-time.

The links between improvisation and language production have been considered by several authors who examine the real-time generation of both music and language (Berliner, 1994; Berkowitz, 2010). Both are guided by a grammar and a set of rules, and learning transfer mechanisms regulate skills development in both. Harrison and Pound (1996) believe that musical improvisation shares certain features and functions with spoken language, and improvisation practice could play a crucial role in improving the ability for speech. Proposing improvisation activities at the developmental level is relevant because it allows one to experience prototypical forms of expression through music.

Regarding its role in musical education, improvisation is often overlooked and relegated to a marginal role in music education curricula, which are mainly based on the development of convergent and performance skills. Several authors claim that improvisation should be a significant part of general education (Azzara, 2002; Campbell, 2009; Hickey, 2009; Wright and Kanellopoulos, 2010; Sawyer, 2011). Burnard (2000, p. 21) notes that “our aim as music educators should be to facilitate a form of music education that focuses on genuine experiences of children *being* improvisers and composers rather than acting out a pre-defined model.” Improvisation offers several advantages that could be used to connect informal and formal learning. Improvisation is considered a technique that allows students to acquire holistic musical training by merging music theory, ear training, and performance in an information-rich context (Campbell, 2009). Parncutt (2006) notes that improvisation is already offered in several undergraduate programs in areas such as jazz, ethnomusicology, early music, organ and music education, but these offerings should be expanded to include all music performance students. The opportunity to study improvisation both theoretically and practically should be considered mandatory when learning an instrument. Moreover, improvisation could be used to revitalize the relationship between music teaching and learning by offering alternative activities for skills development and a more conscious approach to learning. The use of a reflective improvisation practice could stimulate divergent thinking and help develop an awareness about the abilities involved.

## Conclusion

In the current paper, a review of the cognitive research in the field of musical improvisation is proposed. The outlined framework highlights that improvisation is a complex, multidimensional concept that requires several specific skills. Creative and performance acts defined by musical and social constraints are involved in real-time. Possible educational applications include activities based on the development of processes such as anticipation, use of repertoire, emotive communication, feedback, and flow. When designing curriculum activities, the focus should be on the processes that facilitate improvisation rather than the products of improvisation. It is important to construct innovative educational contexts to apply learning approaches such as learning by doing, problem solving, critical thinking, and divergent skills development. Particular attention should be paid to creating a social learning environment, supporting interactive communication, and stimulating students’ intrinsic motivation to learn. Students must be encouraged to be responsible for their own learning by making them aware of their strengths and weaknesses. These procedures require that teachers focus on the quality of the processes that relate to the development of improvisational expertise rather than on the evaluation of learning products. A teacher should stimulate students to socialize the implicit level of their knowledge by providing them the opportunity to share experiences with the group and promoting the analysis of issues. Posing questions on how problems are resolved and alternative ways to achieve meaningful solutions are relevant techniques for activating mechanisms of thinking that induce an increase in the quality of thought. These aspects should stimulate a different way of learning and teaching musical improvisation than the traditional methods by supporting an approach based on reflection. Reflecting on the processes of improvisation enhances metacognitive strategies, which are vital for effective teaching (Biasutti, 2010). In conclusion, this study highlighted several aspects that can be applied in teaching and learning improvisation by proposing a process-based approach in which teaching strategies, reflective practices and divergent thinking play specific, prominent roles.

### Conflict of Interest Statement

The author declares that the research was conducted in the absence of any commercial or financial relationships that could be construed as a potential conflict of interest.

## Footnotes

1 The word lick in American slang literally means “small amount.” It is a familiar term that was initially associated with jazz and blues and later to rock when artists started playing with jazz influences. The definition of lick depends on the type of music: jazz and blues guitarists tend to talk about licks only during an improvisation. In this case, a lick is a small sequence of notes used in combination with others to improvise. A lick could also be a unit of measurement of a solo that can be divided into licks, even if it is an arbitrary process.
